# Delayed Recognition of an Isolated Tear of the Posteromedial Bundle Following Anterior Cruciate Ligament Reconstruction: A Case Report

**DOI:** 10.7759/cureus.88052

**Published:** 2025-07-16

**Authors:** Mahmood Ajawi, Osama Zeidan, Mariam Almaskati, Reema Rajeev, Noor Jaragh, Abdulla Aljowder

**Affiliations:** 1 Orthopedic Surgery, King Hamad University Hospital, Busaiteen, BHR; 2 Medicine, School of Medicine, Royal College of Surgeons in Ireland – Bahrain, Busaiteen, BHR; 3 Orthopedic Surgery, School of Medicine, Royal College of Surgeons in Ireland – Bahrain, Busaiteen, BHR; 4 Orthopedic Surgery, Arabian Gulf University, Manama, BHR

**Keywords:** acl reconstruction, diagnostic arthroscopy, hyperextension injury, internal bracing, knee instability, nonanatomic augmentation, posterior cruciate ligament, posteromedial bundle, pseudo-lachman sign, semitendinosus autograft

## Abstract

Isolated posteromedial bundle (PMB) injuries of the posterior cruciate ligament (PCL) are rare, often subtle, and easily missed on standard clinical tests and imaging. This case report highlights a delayed diagnosis of PMB injury in a 24-year-old male athlete presenting with persistent knee instability eight months following anterior cruciate ligament (ACL) reconstruction. Despite resolution of anterior laxity, the patient exhibited increased hyperextension, a pseudo-Lachman sign, and a tibiofemoral posterior step-off at 10-20° flexion, while 90° posterior drawer testing and MRI were unremarkable. These findings raised suspicion for isolated PMB injury, which was confirmed via diagnostic arthroscopy. The patient underwent nonanatomic PMB augmentation using a semitendinosus autograft with internal bracing. A structured rehabilitation protocol was followed, incorporating PCL-specific bracing, protected range of motion exercises, delayed hamstring activation, and a staged return to activity. At six months, the patient demonstrated full resolution of symptoms, with return to competitive sports by nine months. Validated functional outcome scores confirmed clinical improvement, including an International Knee Documentation Committee subjective score of 92.0 and a Tegner activity scale improvement from 4 (preoperative) to 7 (postoperative), reflecting return to high-level athletic activity. This case emphasizes the importance of recognizing underappreciated clinical signs of isolated PMB injury, particularly following hyperextension trauma. It underscores the limited sensitivity of MRI in chronic or partial PMB tears and advocates for diagnostic arthroscopy when clinical suspicion remains high. Diagnostic arthroscopy should be considered when clinical findings suggest subtle posterior instability, despite normal imaging results. Targeted PMB augmentation can restore extension stability and deliver excellent functional outcomes, even in delayed presentations.

## Introduction

The posterior cruciate ligament (PCL) is the strongest in the knee and plays a crucial role in maintaining posterior tibial stability relative to the femur. Anatomically, it comprises two functional bundles: the anterolateral bundle (ALB) and the posteromedial bundle (PMB). The PMB resists hyperextension forces and contributes to posterior stability during deep flexion, while the ALB dominates in mid-range motion [1-3].

Anterior cruciate ligament (ACL) reconstruction is a standard surgical procedure, particularly in athletes, with a relatively low early complication rate of approximately 1.34% [4]. When persistent instability occurs following ACL reconstruction, common causes include graft laxity, tunnel malposition, or missed meniscal pathology. However, isolated PMB tears are a rare and underrecognized etiology. These injuries can present subtly with signs such as increased hyperextension, pseudo-Lachman, or posterior step-off at 10-20° flexion and may go undetected on standard MRI or posterior drawer testing.

In this report, we describe a rare case of isolated PMB injury diagnosed eight months following ACL reconstruction in a young athlete. We emphasize the diagnostic challenge posed by this injury pattern, the limitations of imaging in chronic cases, and the utility of diagnostic arthroscopy in confirming the lesion. The case also highlights the efficacy of nonanatomic PMB augmentation with internal bracing in restoring knee stability and facilitating return to competitive sports.

## Case presentation

A 24-year-old male athlete presented with persistent right knee pain and subtle instability eight months following ACL reconstruction, which had been performed after a football-related hyperextension injury.

Initial physical examination revealed increased passive hyperextension of the right knee by 10° compared to 5° on the contralateral side and a grade 1+ pivot shift (glide). Valgus and varus stress testing were unremarkable, and posterior drawer testing at 90° showed no increased laxity. The patient exhibited a smooth, coordinated gait.

**Figure 1:**
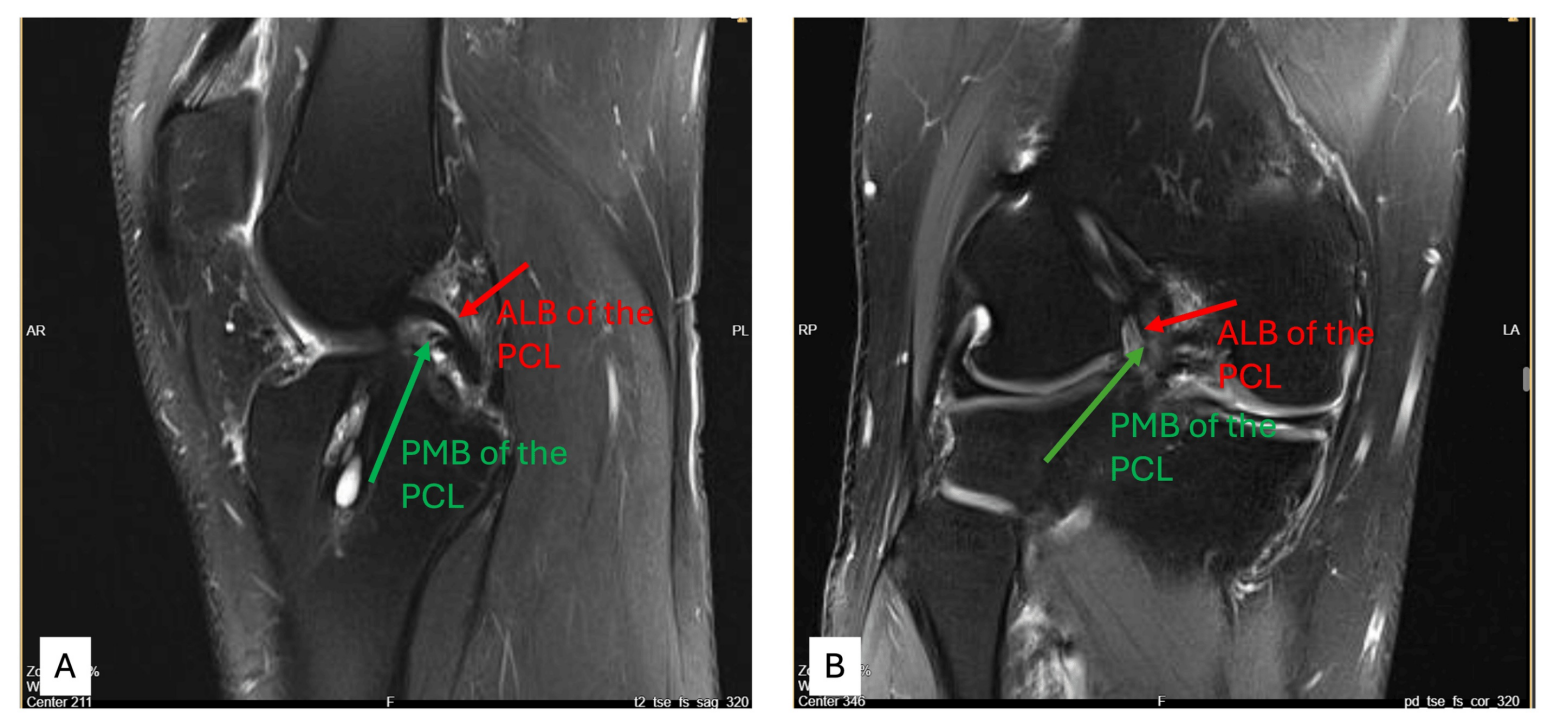
Preoperative MRI demonstrating rupture of the PMB of the PCL (A) Sagittal T2-weighted image showing disrupted and irregular PMB signal. (B) Coronal T2-weighted image confirming loss of continuity in the PMB. MRI: magnetic resonance imaging, PMB: isolated posteromedial bundle, PCL: posterior cruciate ligament, ALB: anterolateral bundle

**Figure 2:**
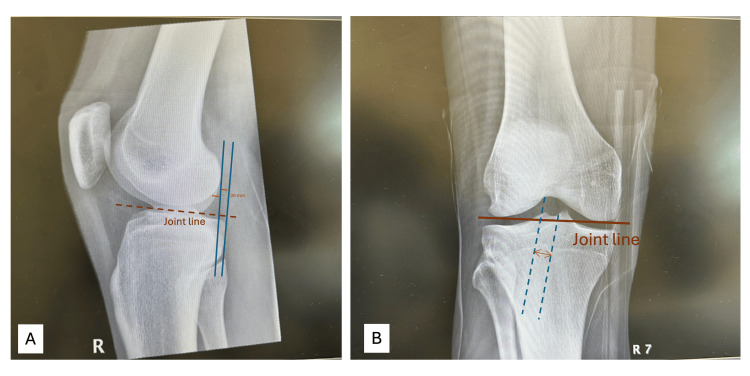
Preoperative X-ray radiographs demonstrating posterior tibial translation (A) Lateral view demonstrating posterior subluxation of the tibia relative to the femur, with a measured posterior translation of approximately 20 mm. (B) Posterior stress AP view showing posterior tibial translation under loading, consistent with posterior instability and isolated PMB rupture. PMB: isolated posteromedial bundle, AP: anteroposterior

Preoperative MRI revealed discontinuity and signal irregularity of the PMB in the sagittal and coronal planes, supporting the clinical suspicion (Figure 1A-1B). Further targeted assessment at 10-20° knee flexion revealed a grade 1+ pseudo-Lachman sign with a firm endpoint, suggestive of anterior-posterior instability at low flexion angles. Additionally, a grade 1+ posterior tibiofemoral step-off was appreciated on posterior drawer testing at 10-20°, despite normal findings at 90°. These findings raised suspicion of isolated PMB insufficiency. The patient underwent PMB augmentation arthroscopically, and preoperative images were obtained (Figure 2A-2B). Objective posterior tibial translation was quantified at 4.1 mm on lateral stress radiography preoperatively, with normalization to <2 mm postoperatively.

**Figure 3:**
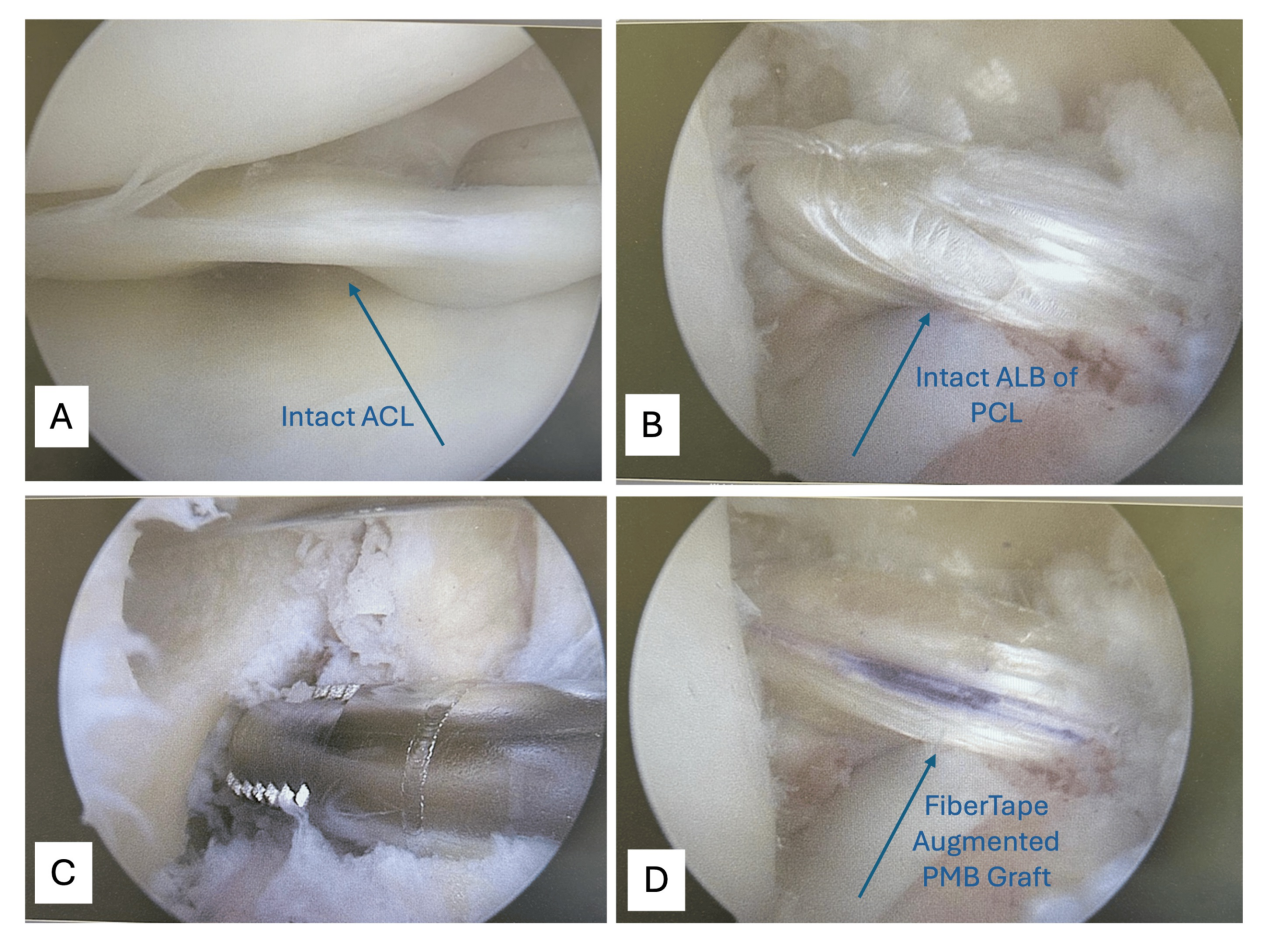
Intraoperative arthroscopic images documenting the diagnostic and surgical steps in isolated PMB augmentation. Findings confirm intact ALB, preserved ACL graft, and successful femoral tunnel creation and graft fixation using internal bracing (A) Intact ACL graft. (B) Intact ALB of the PCL. (C) Femoral tunnel drilling. (D) FiberTape-augmented PMB graft. ACL: anterior cruciate ligament, ALB: anterolateral bundle, PMB: isolated posteromedial bundle, PCL: posterior cruciate ligament

Due to persistent instability and inconclusive imaging, diagnostic arthroscopy was performed. Intraoperative findings confirmed an isolated PMB tear, with preservation of the ALB of the PCL and the ACL graft (Figure 3A-3B). A nonanatomic PMB augmentation was performed using a semitendinosus autograft harvested from the contralateral leg. The graft was fixed with interference screws and reinforced with internal FiberTape bracing and a 4.5 mm PushLock anchor (Figure 3C-3D).

Preoperative radiographs demonstrated subtle posterior tibial translation and disrupted joint alignment, consistent with isolated PMB rupture (Figure 2A-2B). The patient was fitted with a dynamic PCL brace (MediUK) locked in full extension and instructed to remain non-weight-bearing for four weeks. Passive range of motion (ROM) was limited to 0-30° under supervised physiotherapy. At week four, continuous passive motion (CPM) was initiated and gradually progressed to 60° over a two-week period. Hamstring activation was avoided explicitly for six weeks to minimize posterior shear forces and protect the healing PMB graft, as recommended by recent PCL rehabilitation protocols [5]. Quadriceps strengthening and anterior chain loading were emphasized during this phase.

By week 6, the patient began brace weaning and initiated partial weight-bearing. From weeks 6 to 10, proprioceptive exercises and low-impact strength training were introduced. Running and plyometric activity resumed after week 16, contingent upon normalized strength and joint stability.

Notably, the preoperative grade 1+ pivot shift, despite an intact ACL graft, was attributed to rotational instability resulting from PMB deficiency. In this case, the pivot shift was likely a manifestation of altered tibiofemoral mechanics related to posterior instability at low flexion angles. Following PMB augmentation, the pivot shift resolved completely, supporting the biomechanical role of the PMB in rotational control.

At the six-month follow-up, the patient exhibited complete resolution of preoperative instability, negative pivot and drawer tests, and objectively documented clinical improvement. He returned to competitive sports at nine months postoperatively. Table 1 shows a summary of the preoperative and postoperative clinical findings. Additionally, the patient's International Knee Documentation Committee (IKDC) subjective knee evaluation score, a validated patient-reported outcome measure, improved from 52.9 preoperatively to 91.95 at six-month follow-up, reflecting marked improvement in knee function and perceived quality of recovery. The Tegner activity scale score improved from 4 preoperatively (light recreational activity) to 7 postoperatively (return to competitive sports), indicating restoration of high-level physical function.

**Table 1:** Summary of preoperative and postoperative clinical findings MRI: magnetic resonance imaging, IKDC: International Knee Documentation Committee, PCL: posterior cruciate ligament, N/A: not applicable

Parameter	Preoperative findings	Postoperative findings
Passive hyperextension (°)	10° on the right side vs. 5° contralateral	Resolved
Pseudo-Lachman (10–20°)	Positive (grade 1+)	Negative
Tibiofemoral posterior step-off (10–20°)	Grade 1+	Resolved
Pivot shift test	Grade 1+ glide	Negative
Posterior drawer at 90°	Normal	Normal
MRI findings	No definitive PCL injury	Not repeated
Gait	Normal	Normal
Return to sports	N/A	Yes, at 9 months
IKDC score	52.9	91.95
Tegner activity scale	4	7

## Discussion

Interpretation of findings

This case illustrates the diagnostic and therapeutic challenges of isolated PMB injuries of the PCL, which are rare, subtle, and often missed, particularly when subtle symptoms arise following ACL reconstruction. The patient, a 24-year-old male athlete, presented with right knee instability and discomfort eight months postoperatively, following a hyperextension injury during a football match. While anterior stability was restored following ACL reconstruction, key residual signs were observed: a grade 1+ pseudo-Lachman sign at 10-20° of knee flexion, 10° passive hyperextension (vs. 5° contralateral), and a grade 1+ tibiofemoral posterior step-off at the same range. These findings, despite a normal posterior drawer test at 90°, highlight how standard assessments may miss isolated PMB tears.

This constellation of findings is indicative of an isolated PMB injury of the PCL. The PMB functions primarily in full extension and deep flexion, providing posterior tibial restraint in these positions, whereas the ALB is most active in mid-flexion (30-90°) [6]. Standard PCL assessment methods primarily evaluate ALB integrity and may overlook isolated PMB tears.

Recent case series have described a triad of findings in isolated PMB injuries: (1) pseudo-Lachman at 10-20° flexion, (2) posterior step-off in the same range, and (3) increased passive hyperextension compared to the contralateral limb. These signs, all present in this patient, were consistently observed in 12 patients in a landmark study by Mouton et al. [7], identifying this constellation as a unique clinical entity following hyperextension trauma.

Of particular interest is the presence of a grade 1+ pivot shift, typically pathognomonic for ACL insufficiency. In this case, however, the ACL graft was intact. This suggests a biomechanical link between isolated PMB deficiency and subtle rotational laxity, possibly due to altered posterior tibial alignment at terminal extension and low flexion angles. PMB insufficiency can affect tibiofemoral kinematics, leading to a misleading pivot shift phenomenon. The misleading pivot shift was eliminated following PMB augmentation, further supporting this biomechanical interpretation.

MRI was inconclusive preoperatively, a common limitation in diagnosing partial or chronic PCL tears. PMB injuries may heal with fibrosis or exhibit signal normalization over time, leading to false negatives [7]. This reinforces the diagnostic utility of detailed examination at specific flexion angles and stress radiography, as well as the need for diagnostic arthroscopy when clinical suspicion remains high [8]. The under-recognition of PMB injuries in the literature has led to diagnostic delays, particularly in cases presenting with subtle signs and normal imaging. This case highlights the importance of thorough, multi-angle clinical evaluation and underscores the role of arthroscopy in confirming diagnoses. Successful management through targeted PMB reconstruction adds to a growing body of evidence advocating for specific identification and treatment of PMB injuries as a distinct subset.

Surgical technique

The decision to proceed with isolated PMB augmentation was based on arthroscopic confirmation of PMB rupture and preservation of the ALB and ACL graft. A nonanatomic PMB augmentation was performed using a semitendinosus autograft reinforced with internal FiberTape bracing. This technique allows for the selective restoration of the deficient bundle while minimizing the risk of tunnel convergence and preserving native structures, as advocated by Ibáñez et al. [8]. Internal bracing provides early mechanical stability, supports graft integration, and facilitates a more accelerated rehabilitation timeline, which is advantageous in athletic patients.

Rehabilitation and clinical implications

A carefully staged rehabilitation protocol was employed to protect the graft and promote optimal healing. During the initial four-week phase, the patient remained non-weight-bearing with a dynamic PCL brace locked in full extension. This alignment reduces posterior tibial sag, a known cause of graft laxity [6]. Passive ROM was limited to 0-30° under physiotherapy guidance.

CPM was initiated at week 4 and increased gradually to 60° over a period of two weeks. Hamstring activation was avoided for six weeks to reduce posterior tibial translation and shear forces, which can compromise the graft in early healing [5]. Instead, emphasis was placed on quadriceps strengthening to promote anterior tibial translation and stabilize the knee.

By weeks 6-10, partial weight-bearing and controlled ROM were introduced. Proprioceptive training and closed kinetic chain exercises were progressively incorporated, followed by low-impact cardiovascular training. Running and plyometrics began only after 16-20 weeks, contingent on achieving strength symmetry and joint stability [9]. Return to unrestricted sports participation occurred at nine months postoperatively, following comprehensive functional testing. This timeline reflects best practice standards in isolated PMB rehabilitation [5]. No interim complications were reported during the follow-up period.

Clinically, this case highlights a vital takeaway: persistent instability following ACL reconstruction is not always indicative of graft failure. In the absence of anterior laxity, clinicians must consider alternative diagnoses such as isolated PMB insufficiency. The classic triad of signs, pseudo-Lachman, posterior step-off at 10-20°, and hyperextension asymmetry, may be pathognomonic. MRI has limited sensitivity in these cases due to the masking of the lesion by scar tissue [10].

When suspicion persists despite inconclusive imaging, diagnostic arthroscopy remains the definitive tool. Isolated PMB injuries can be successfully treated with nonanatomic augmentation and internal bracing, providing excellent biomechanical outcomes without compromising other cruciate elements [11].

## Conclusions

Isolated PMB injuries of the PCL are an underrecognized source of knee instability, particularly after ACL reconstruction. Subtle signs such as hyperextension asymmetry, pseudo-Lachman at 10-20° flexion, and a posterior step-off should raise clinical suspicion, even when the 90° posterior drawer test and MRI appear normal. In such cases, diagnostic arthroscopy remains essential to confirm the lesion.

Our case highlights the importance of maintaining clinical vigilance in post-ACL reconstruction patients with unresolved instability despite an intact graft. Early recognition and targeted surgical management, specifically nonanatomic PMB augmentation with internal bracing, can restore knee function, prevent prolonged disability, and support full return to sport, as demonstrated by the patient’s recovery within nine months following structured rehabilitation. Furthermore, this case highlights the need to establish a standardized diagnostic algorithm for PMB injuries and encourages future research on the long-term outcomes of this surgical technique.
